# dbPSP: a curated database for protein phosphorylation sites in prokaryotes

**DOI:** 10.1093/database/bav031

**Published:** 2015-04-03

**Authors:** Zhicheng Pan, Bangshan Wang, Ying Zhang, Yongbo Wang, Shahid Ullah, Ren Jian, Zexian Liu, Yu Xue

**Affiliations:** ^1^School of Life Sciences, University of Science and Technology of China, Hefei 230027, China, ^2^Department of Biomedical Engineering, College of Life Science and Technology, Huazhong University of Science and Technology, Wuhan, Hubei 430074, China, and ^3^State Key Laboratory of Biocontrol, School of Life Sciences, School of Advanced Computing, Sun Yat-sen University, Guangzhou 510275, China

## Abstract

As one of the most important post-translational modifications, phosphorylation is highly involved in almost all of biological processes through temporally and spatially modifying substrate proteins. Recently, phosphorylation in prokaryotes attracted much attention for its critical roles in various cellular processes such as signal transduction. Thus, an integrative data resource of the prokaryotic phosphorylation will be useful for further analysis. In this study, we presented a curated database of phosphorylation sites in prokaryotes (dbPSP, Database URL: http://dbpsp.biocuckoo.org) for 96 prokaryotic organisms, which belong to 11 phyla in two domains including bacteria and archaea. From the scientific literature, we manually collected experimentally identified phosphorylation sites on seven types of residues, including serine, threonine, tyrosine, aspartic acid, histidine, cysteine and arginine. In total, the dbPSP database contains 7391 phosphorylation sites in 3750 prokaryotic proteins. With the dataset, the sequence preferences of the phosphorylation sites and functional annotations of the phosphoproteins were analyzed, while the results shows that there were obvious differences among the phosphorylation in bacteria, archaea and eukaryotes. All the phosphorylation sites were annotated with original references and other descriptions in the database, which could be easily accessed through user-friendly website interface including various search and browse options. Taken together, the dbPSP database provides a comprehensive data resource for further studies of protein phosphorylation in prokaryotes.

**Database URL:** http://dbpsp.biocuckoo.org

## Introduction

As one of the most ubiquitous and important protein post-translational modifications (PTMs), the reversible protein phosphorylation was involved in almost all biological processes (1, 2). Phosphorylation was catalysed by a protein kinase through transferring a phosphate moiety from adenosine triphosphates (ATPs) to the acceptor residue in the substrate (2). Phosphorylation in eukaryotes was extensively studied during the past decades since 1932 (3), and most of the identified phosphorylation acceptor residues were serine (Ser), threonines (Thr) and tyrosines (Tyr) (4, 5). Protein phosphorylation had been regarded as a biological process exclusively in eukaryotes until the first evidence of the phosphorylation in bacteria, which was identified in isocitrate dehydrogenase from *Escherichia coil* by Garnak and Reeves (6) in 1979, while protein phosphorylation in archaea was reported in the extreme halophilic archaeon *Halobacterium salinarum* by Spudich and Stoeckenius (7) in 1980. Subsequently, phosphorylation in prokaryotes were extended to other residues such as histidine (His) (8), aspartic acid (Asp) (9) and cysteine (Cys) (10). It was found that His/Asp phosphorylation plays critical roles in various cellular processes such as two-component system based signaling transduction (11), while Ser/Thr/Tyr phosphorylation in prokaryotes attracted more and more attention recently (12).

Recently, rapid progresses in high-throughput (HTP) mass spectrometry based proteomic technologies greatly advanced the identification of phosphorylation sites (13, 14). Numerous studies have been carried out to profile the phosphorylation events and advance the phosphoproteome techniques to a state-of-the-art stage (13, 14). For example, recently Sharma *et al*. (15) identified over 30 000 phosphorylation events in a single human cancer cell line. Although only a handful studies have been contributed to the large-scale identification of phosphorylation in prokaryotes in comparison with eukaryotes, outstanding progresses were made by leading scientists. For example, Macek *et al*. (16) profiled 78 phosphorylation sites by high-accuracy mass spectrometry and biochemical enrichment of phosphopeptides from model bacterium *Bacillus subtilis* in 2007, and further detected 81 phosphorylation sites from the model Gram-positive bacterium *Escherichia coli* in 2008 (17)*.* Recently, 410 phosphorylation sites from 245 proteins Ming-kun were identified in *Synechococcus* sp.* PCC 7002* by Yang *et al*. (18), while Reimann *et al*. (19) detected 801 phosphoproteins in *Sulfolobus solfataricus*. Besides Ser/Thr/Tyr phosphorylation, Elsholz *et al*. (20) profiled 121 arginine (Arg) phosphorylation sites in 87 proteins from *B.* *subtilis in vivo*. These leading studies made great contributions to expanding the understanding of molecular mechanisms and functional roles for phosphorylation in prokaryotes.

As the discoveries accumulated, the collection and maintenance of the identified phosphorylation sites became an urgent issue to be solved. Previously, a number of comprehensive databases for phosphorylation sites were constructed (21), while most of which were focused on eukaryotes. Databases such as Phosphorylation Site Database (22), SysPTM 2.0 (23), PHOSIDA (24), dbPTM 3.0 (25) and UniProt (26) have collected the prokaryotic phosphorylation sites. However, only a limited proportion of the identified prokaryotic phosphoproteins and sites were covered. In this study, we developed and presented the database of phosphorylation sites in prokaryotes (dbPSP). Totally, 7391 phosphorylation sites on seven types of phosphorylated residues including serine (Ser), threonine (Thr), tyrosine (Tyr), aspartic acid (Asp), histidine (His), cysteine (Cys) and arginine (Arg) in 3750 prokaryotic proteins from 96 organisms in 11 phyla were manually curated from the published literature. On the basis of the datasets, we analysed the sequence preferences of the phosphorylation sites and functional annotations of the phosphoproteins among eukaryotes, bacteria and archaea, while the results show that there were obvious differences among phosphorylation in the three domains of life. Taken together, the dbPSP database could serve as a comprehensive data resource for further studies of protein phosphorylation in prokaryotes.

## Construction and content

**Figure 1.**
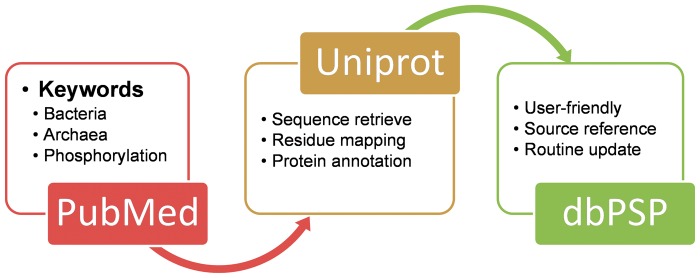
The schema of the construction processes and contents for the dbPSP database.

The construction of database dbPSP was summarized as a diagram in Figure 1A. We searched PubMed (http://www.ncbi.nlm.nih.gov/pubmed) with keywords including ‘bacteria phosphorylation’, ‘archaea phosphorylation’ and ‘archaebacteria phosphorylation’ (1 March 2014). All the retrieved 16 658 articles were manually reviewed and checked by domain experts to collect the experimentally identified prokaryotic phosphorylation sites. The curated phosphorylated residues were explicitly mapped to UniProt proteomes sequences (Release 2014_06) (26), while the annotations and cross references of phosphoproteins were also retrieved from UniProt database and integrated into the database. The references which identified phosphorylation sites were also provided in the dbPSP database.

Besides manual curation from literatures, the prokaryotic phosphorylation sites in public databases were also collected. From databases including PHOSPHORYLATION SITE DATABASE (22), SysPTM 2.0 (23), PHOSIDA (24), dbPTM 3.0 (25) and UniProt (26), 1400, 348, 305, 186 and 176 phosphorylation sites were retrieved, respectively (Table 1). These datasets were cross-checked with our manually collected dataset and integrated into dbPSP database. In total, 7391 non-redundant phosphorylation sites among seven types of residues were found in 3750 substrates from 11 phyla were provided in the database, which present a comprehensive data resource for prokaryotic phosphorylation. In total, 7171 and 209 sites were identified by HTP and low-throughput studies, respectively. Various annotations such as protein names, gene names, keywords, functional descriptions and sequence annotations from the UniProt database (26) were retrieved to annotate the collected phosphoproteins.

**Table 1.** The comparison for the numbers of prokaryotic phosphorylation sites among dbPSP and other databases

Database	Sites	Phosphoproteins	Articles
dbPSP	7391	3750	174
Phosphorylation Site Database	1400	960	—
SysPTM 2.0	348	213	7
PHOSIDA	305	282	4
dbPTM 3.0	186	138	54
UniProt	176	135	73

-, Phosphorylation Site Database is not available.

**Figure 2.**
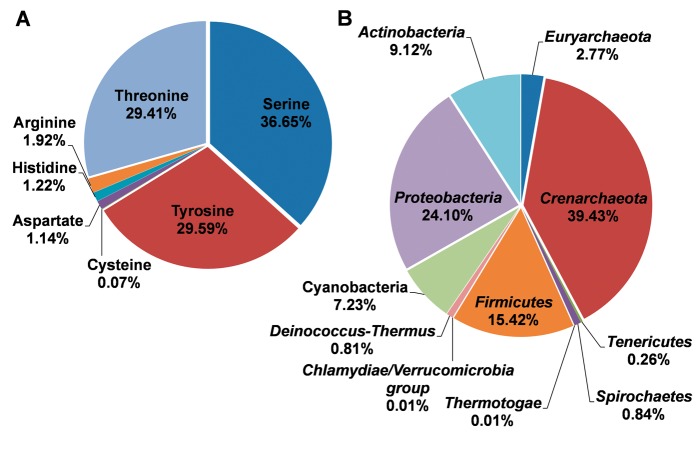
The distributions of residues types and species for the phosphoproteins in prokaryotes. (**A**) The distributions of residues types. (**B**) The distribution of phyla.

With the abundant phosphorylation sites, the distributions for different residue types and species were summarized, while the results were presented in Figure 2. It was observed that phosphorylated serine, tyrosine, and threonine occupied 36.65%, 29.59% and 29.41% of modified residues, respectively (Figure 2A). The known phosphorylation sites on aspartic acid, histidine, cysteine, and arginine were limited and need further studies to explore (Figure 2A). In the dbPSP database, the phosphorylation sites were collected from 96 prokaryotic organisms in 11 phyla. The distribution of species at the phyla level was presented in Figure 2B. The phylum *Crenarchaeota* and *Proteobacteria* have the most substrates with the most proportions of 39.43% and 24.10%, respectively (Figure 2B), while phosphorylation sites in Thermotogae and *Chlamydiae/*Verrucomicrobia group were limited.

## Usage

**Figure 3.**
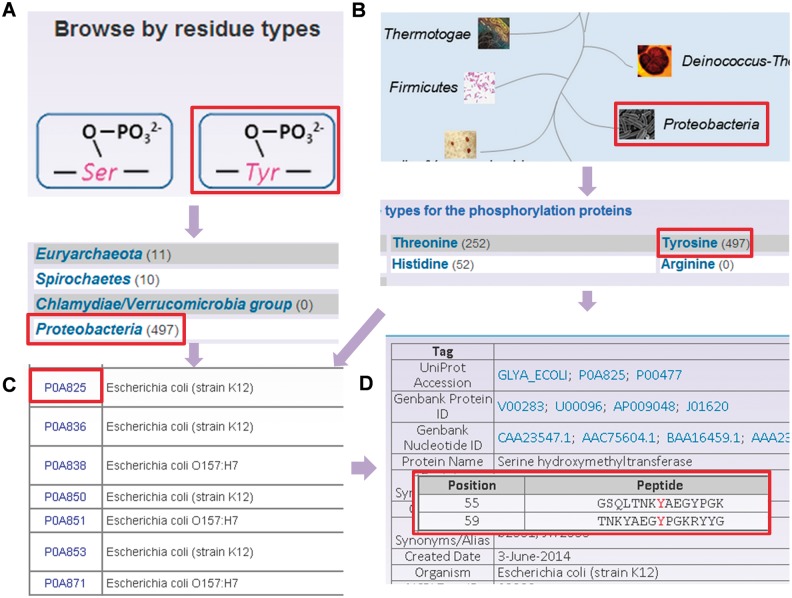
The browse options of dbPSP database. (**A**) Browse option by residue types. (**B**) Browse option by phyla. (**C**) The tyrosine phosphorylated phosphoprotein list in. (**D**) The detailed information of phosphorylated serine hydroxymethyltransferase from *E. coli* (strain K12).

To provide convenient usage, the database was developed in a user-friendly manner, while browse and search options were provided to access the information of prokaryotic phosphorylation sites in the database. Since the phosphorylation sites are identified in different residues and various species, two browse options including ‘Browse by residue types’ (Figure 3A) and ‘Browse by phyla’ (Figure 3B) were developed in the database. Here, the serine hydroxymethyltransferase in *E. coli* (strain K12) was selected as an example to describe the usage of browse and search options. In the ‘Browse by residue types’, the phosphorylated residues are shown in diagrams (Figure 3A). By clicking the diagram of tyrosine phosphorylation, the distribution of tyrosine phosphorylated phosphoproteins in various organisms is returned (Figure 3A). Then the tyrosine phosphorylated phosphoproteins in *Proteobacteria* could be listed in a tabular format with ‘UniProt Accession’, ‘Name/Alias’ by clicking the link of ‘*Proteobacteria’* (Figure 3C). In the option of ‘Browse by phyla’ (Figure 3B), the 11 phyla in two domains including bacteria and archaea are listed for users to browse the phosphoproteins (Figure 3C). Through clicking on the figure of ‘*Proteobacteria’*, the distribution of phosphoproteins for different modification residue types is shown (Figure 3C). Then the list of tyrosine phosphorylated phosphoproteins could be retrieved after clicking the link ‘Tyrosine’, while the detailed information for specific phosphoproteins is provided by clicking protein entry (Figure 3D).

**Figure 4.**
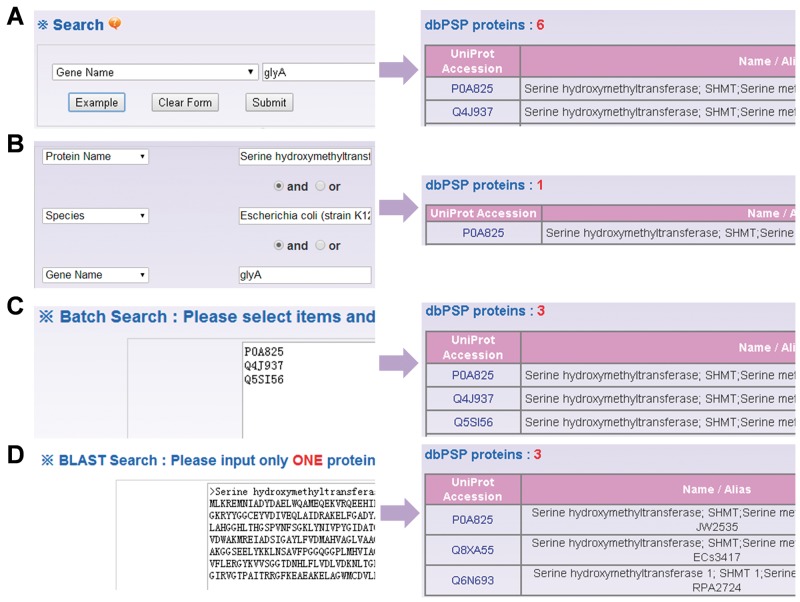
The search options of dbPSP database. (**A**) The database could be searched by simple key words. (**B**) The ‘Advanced Search’ allowed users to submit up to three terms for search. (C) The ‘Batch Search’ for retrieving multiple protein entries with a list of terms. (**D**) The database could be queried with a protein sequence to find identical or homologous phosphoproteins.

Besides browse options, the web interface provides four search options including simple search (Figure 4A), ‘Advanced Search’ (Figure 4B), ‘Batch Search’ (Figure 4C) and ‘Blast Search’ (Figure 4D). For example, if user input the keyword ‘glyA’ in the ‘Gene Name’ area, the results will be generated in a tabular format with ‘UniProt Accession’, ‘Name/Alias’ (Figure 4A). Alternatively, users can use the ‘Advanced Search’ with three search terms specified in different areas and combined with three operators of ‘and’, ‘or’ and ‘exclude’, which could reduce the potential hits and provide highly related results (Figure 4B). Furthermore, ‘Batch Search’ is designed for retrieving multiple phosphoproteins with a list of keywords (Figure 4C). Finally, ‘Blast Search’ is implemented in the database to find homologous proteins with a protein sequence in Fasta Format. The NCBI BLAST package (27) is employed search related sequences (Figure 4D).

## Discussion

As one of most important protein PTMs, prokaryotic protein phosphorylation was critical for numerous cellular processes through modification of various types of residues (28, 29). After the first discovery of phosphorylation events in prokaryotes, a large number of substrates and sites have been identified to dissect the molecular mechanisms and functional roles of phosphorylation. Although previously various databases were developed to maintain the known phosphorylation sites, most of these databases were focused on eukaryotes. In this regard, an integrated and comprehensive database for prokaryotic phosphorylation is urgently needed. In this study, we presented a manually curated and comprehensive database of dbPSP, which aimed to maintain known phosphorylation sites from various organisms in prokaryotes.

**Figure 5.**
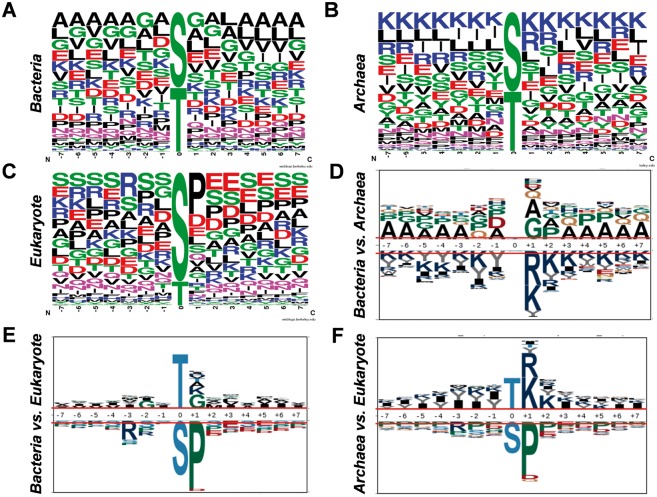
Analyses of sequence preferences of phosphorylation sites in prokaryotes. The sequence preferences of phosphorylation sites in bacteria (**A**), archaea (**B**) and eukaryotes (**C**) were presented with WebLogo. The comparisons of sequence preferences for bacteria and archaea (**D**), bacteria and eukaryotes (**E**), archaea and eukaryotes (**F**).

Previously, numerous studies on eukaryotes indicated that phosphorylation was mediated by linear motifs (5, 30). With the dataset collected in this study, we analysed the sequence preferences and motifs for Ser/Thr phosphorylation in bacteria (Figure 5A), archaea (Figure 5B) and eukaryotes (Figure 5C), while 10 092 eukaryotic Ser/Thr phosphorylation sites from phospho.ELM database were employed for comparison (31). As the sequence preferences illustrated by WebLogo (32), alanine and lysine has high frequencies around the phosphorylation sites in bacteria (Figure 5A) and archaea (Figure 5B), respectively, there were abundant serine and glutamic acid around the phosphorylated residues (Figure 5C). To further dissect the differences, pLogo was employed to pairwisely compare the sequence preferences (Figure 5D–F) (33). It was observed that positively charged residues including arginine and lysine were enriched around phosphorylated Ser/Thr in archaea than bacteria (Figure 5D) and eukaryotes (Figure 5F), while proline were over-presented in +1 position of the phosphorylation sites in eukaryotes than bacteria (Figure 5E) and archaea (Figure 5F). Taken together, obvious differences were observed among the sequence preferences of phosphorylation sites in the three domains of organisms.

**Figure 6.**
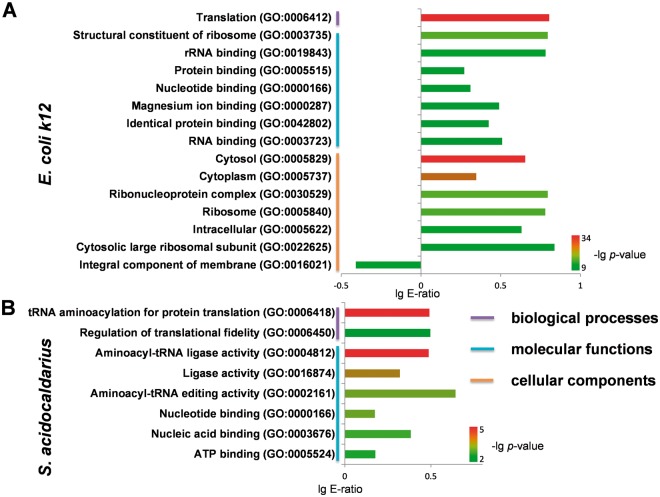
Statistical analyses of GO annotation for phosphoproteins in *E. Coli k12* and *S. acidocaldarius. *(**A**) The enriched GO terms for phosphoproteins in *E. Coli k12*. (**B**) The enriched GO terms for phosphoproteins in *S. acidocaldarius*.

Furthermore, with the comprehensive phosphorylation datasets in the dbPSP database, we tried to analyze the functional annotations of phosphoproteins in prokaryotes with the examples of *E.* *coli* (strain K12) (*E. Coli k12*) and *Sulfolobus acidocaldarius*, which contained the most identified phosphoproteins and sites in bacteria and archaea, respectively. The gene ontology (GO) (31 March 2012) association files were downloaded from the The Gene Ontology Annotation (GOA) database at the European Bioinformatics Institute (EBI) (http://www.ebi.ac.uk/goa) (34) and the complete proteomes were retrieved from AmiPro Database (26). With hypergeometric distribution (35), we statistically analysed the enriched biological processes, molecular functions and cellular components for phosphoproteins in *E. Coli k12* (Figure 6A, *P-*value < 10^−^^9^) and *S. acidocaldarius* (Figure 6B, *P-*value < 10^−^^2^). It was observed that translation (GO:0006412) was the intensively enriched biological process in phosphoproteins from *E. Coli k12* (Figure 6A), while translation-related annotations of tRNA aminoacylation for protein translation (GO:0006418) and regulation of translational fidelity (GO:0006450) were also over-presented in phosphoproteins from *S. acidocaldarius* (Figure 6). For molecular functions, phosphoproteins from *E. Coli k12* and *S. acidocaldarius* both enriched annotations of nucleotide binding (GO:0000166) (Figure 6). Furthermore, phosphoproteins from *E. Coli k12* over-presented other molecular functions including structural constituent of ribosome (GO:0003735), rRNA binding (GO:0019843), protein binding (GO:0005515), magnesium ion binding (GO:0000287), identical protein binding (GO:0042802) and RNA binding (GO:0003723) (Figure 6A), while phosphoproteins from *S. acidocaldarius* enriched aminoacyl-tRNA ligase activity (GO:0004812), ligase activity (GO:0016874), aminoacyl-tRNA editing activity (GO:0002161), nucleic acid binding (GO:0003676) and ATP binding (GO:0005524) (Figure 6B). In addition, a handful of cellular components were over-presented in phosphoproteins from *E. Coli k12* (Figure 6A), while no enrichment was observed in for *S. acidocaldarius*.

Taken together, in this study the dbPSP database was developed to maintain the experimentally identified phosphorylation sites in prokaryotes. We anticipated that such database could provide a useful resource for further studies and understanding of phosphorylation in prokaryotes.

## Funding

This work was supported by grants from the National Basic Research Program (973 project) (2013CB933900 and 2012CB910101); Natural Science Foundation of China (31171263, 81272578 and J1103514); International Science and Technology Cooperation Program of China (2014DFB30020); and China Postdoctoral Science Foundation (2014M550392). Funding for open access charge: 31171263.

*Conflict of interest*. None declared.
